# A randomised dose-ranging study of tiotropium Respimat® in children with symptomatic asthma despite inhaled corticosteroids

**DOI:** 10.1186/s12931-015-0175-9

**Published:** 2015-02-07

**Authors:** Christian Vogelberg, Petra Moroni-Zentgraf, Migle Leonaviciute-Klimantaviciene, Ralf Sigmund, Eckard Hamelmann, Michael Engel, Stanley Szefler

**Affiliations:** University Hospital Carl Gustav Carus, Technical University of Dresden, Fetscherstraße 74, 01307 Dresden, Germany; Boehringer Ingelheim Pharma GmbH & Co. KG, Ingelheim am Rhein, Germany; Vilnius University Hospital, Vilnius, Lithuania; Boehringer Ingelheim Pharma GmbH & Co. KG, Biberach an der Riss, Germany; Evangelisches Krankenhaus Bielefeld, Bielefeld, Germany; Department of Pediatrics, Children’s Hospital of Colorado and the University of Colorado Denver School of Medicine, Aurora, Colorado USA

**Keywords:** Asthma, Asthma control, Children, Once-daily, Tiotropium, Lung function, Paediatric, Respimat®

## Abstract

**Background:**

A considerable number of children with asthma remain symptomatic despite treatment with inhaled corticosteroids, resulting in significant morbidity, reduced quality of life, increased healthcare costs and lost school days. The aim of our study was to assess the efficacy, safety and tolerability of once-daily tiotropium Respimat® 5 μg, 2.5 μg and 1.25 μg add-on to medium-dose inhaled corticosteroids, with or without a leukotriene modifier, in children aged 6–11 years with symptomatic asthma.

**Methods:**

In this Phase II, double-blind, placebo-controlled, incomplete-crossover, dose-ranging study, patients were randomised to receive three of the four treatments evaluated: once-daily tiotropium Respimat® 5 μg, 2.5 μg or 1.25 μg or placebo Respimat®, in the evening during the 12-week (three × 4-week) treatment period.

**Results:**

In total, 76, 74, 75 and 76 patients aged 6–11 years received tiotropium Respimat® 5 μg, 2.5 μg, 1.25 μg and placebo Respimat®, respectively. For the primary end point (peak forced expiratory volume in 1 second measured within 3 hours post-dosing), the adjusted mean responses with tiotropium Respimat® 5 μg (272 mL), 2.5 μg (290 mL) and 1.25 μg (261 mL) were significantly greater than with placebo Respimat® (185 mL; p = 0.0002, p < 0.0001 and p = 0.0011, respectively). The safety and tolerability of all doses of tiotropium Respimat® were comparable with those of placebo Respimat®, with no serious adverse events and no events leading to discontinuation.

**Conclusions:**

Tiotropium Respimat® add-on to medium-dose inhaled corticosteroids, with or without a leukotriene modifier, was efficacious in paediatric patients with symptomatic asthma and had comparable safety and tolerability with placebo Respimat®.

**Trial registration:**

ClinicalTrials.gov identifier NCT01383499

**Electronic supplementary material:**

The online version of this article (doi:10.1186/s12931-015-0175-9) contains supplementary material, which is available to authorized users.

## Background

Asthma is a leading cause of childhood morbidity [1]. In the USA alone, around 7 million children and adolescents suffer from asthma [2], and in the UK, one in every seven children aged 2–15 years has asthma symptoms requiring regular treatment [3]. As in adults, a considerable proportion of asthma in children is inadequately controlled by inhaled corticosteroid (ICS) guideline therapy, which represents a significant healthcare concern [4]. In addition to the negative impact on patients’ quality of life, this considerably increases their risk of future exacerbations, with associated increased requirement for healthcare utilisation and costs [5-7]. Data for the USA show that in 2011, 56% of children with asthma suffered an attack [2], with almost 20% visiting an emergency department [8].

While the goals of treatment for children with asthma [9,10] are broadly the same as for adults (to improve control, reduce exacerbations, reduce rescue medication usage, reduce hospitalisations and allow maximum possible participation in normal daily activities), treatment can be complicated by issues that are specific to, or more pronounced in, this age group. Adherence to asthma medication is notably poor in children and adolescents [11], and compliance with twice-daily ICS treatment regimens may be sub-optimal, particularly during asymptomatic periods. In addition, the detection and appropriate management of children with poor asthma control is hampered by the fact that both the affected children and their parents tend to underestimate their asthma severity [5,6,11-14].

Given the prevalence of uncontrolled asthma in children and its health, educational and financial impact, there is clearly a need to further improve asthma control and prevent exacerbations in this population; the long-acting anticholinergic bronchodilator tiotropium represents a potential add-on therapy for such patients [15,16]. Tiotropium has demonstrated efficacy in the treatment of asthma in adults [17-21] and adolescents [22]. Here we report data from the first assessment of tiotropium treatment in children aged 6–11 years with symptomatic asthma. This study evaluated the efficacy, safety and tolerability of three doses of tiotropium in children with symptomatic asthma despite maintenance treatment with ICS.

## Methods

### Study design

This Phase II, randomised, double-blind, placebo-controlled, incomplete-crossover, dose-ranging study was conducted at 24 centres in six countries from 23 August 2011 to 25 September 2012. The study met all local legal and regulatory requirements and conformed to the Declaration of Helsinki and to Good Clinical Practice and Good Publication Practice guidelines. The protocol was approved by an independent ethics committee at each study centre, and all patients and their parents or legal guardians provided written, informed consent.

**Figure 1:**
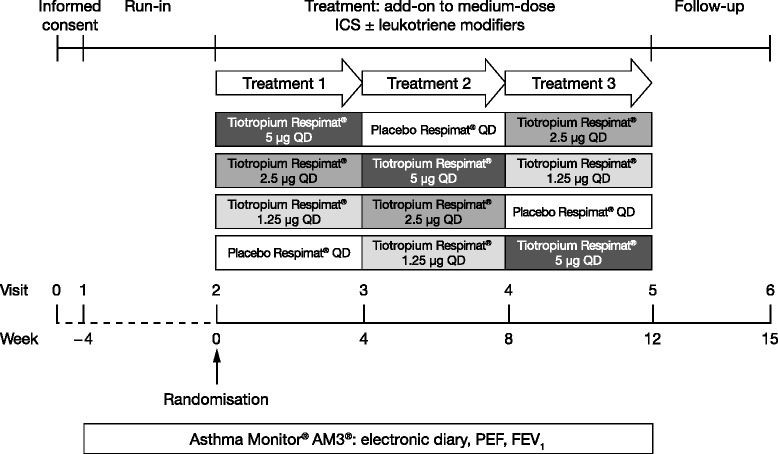
**Study design.** FEV_1_, forced expiratory volume in 1 second; ICS, inhaled corticosteroids; PEF, peak expiratory flow; QD, once-daily.

Following a 4-week run-in period, during which patients received ICS maintenance therapy with or without a leukotriene modifier, patients were randomised in a 1:1:1:1 ratio to receive once-daily tiotropium 5 μg, 2.5 μg, 1.25 μg or placebo, all delivered via the Respimat® SoftMist™ inhaler (Boehringer Ingelheim Pharma GmbH & Co. KG, Ingelheim am Rhein, Germany), during three 4-week treatment periods. Patients received three of the four available treatments with no washout between treatment periods (Figure 1) as pharmacodynamic steady state with tiotropium is known to be achieved after 3 weeks in patients with chronic obstructive pulmonary disease [23,24]. All study treatments (tiotropium Respimat® 5 μg, 2.5 μg, 1.25 μg and placebo Respimat®) were self-administered, under parental supervision every evening, double-blind as add-on to maintenance treatment with medium-dose ICS (200–400 μg budesonide or equivalent dose), with or without a leukotriene modifier. Patients and parents received training on the use of the Respimat® SoftMist™ inhaler at Visits 1 (screening) and 2 (randomisation), and at later visits if required. Blinding was maintained up to database lock. Rescue medication (open-label salbutamol inhaler, 100 μg per puff) was permitted during screening and the entire treatment period. The use of antibiotics was not restricted during the trial; temporary increases in the dose of ICS or addition of systemic steroids was permitted as well as the addition of short-acting theophylline preparations for the treatment of acute exacerbations.

A fixed block randomisation was used to ensure that a balanced number of patients was allocated to each treatment. The order of patient assignment to treatment sequences was randomised. The randomisation list was generated by Boehringer Ingelheim using a validated system with a pseudo-random number generator and supplied seed number.

### Study population

Male and female patients aged 6–11 years with a ≥6-month history of asthma and diagnosis confirmed at screening were eligible for enrolment into this study. All patients were required to have bronchodilator reversibility resulting in a forced expiratory volume in 1 second (FEV_1_) increase of ≥12% within 15–30 minutes after the administration of 200 μg salbutamol, and to be symptomatic at screening and prior to randomisation, as defined by a seven-question Asthma Control Questionnaire (ACQ-7) mean score of ≥1.5. All patients received maintenance therapy with ICS at a stable medium dose (200–400 μg budesonide or equivalent dose) either as monotherapy or in combination with a long-acting β_2_-agonist (LABA) or leukotriene modifier for ≥4 weeks prior to screening (LABAs had to be stopped at least 24 hours prior to screening; however, leukotriene modifiers were permitted throughout the trial). In addition, all patients had to have a pre-bronchodilator FEV_1_ 60–90% of predicted normal at screening and were to demonstrate pre-bronchodilator FEV_1_ variability at randomisation within ±30% compared with the screening value. Exclusion criteria included a significant medical condition other than asthma, congenital heart disease, any acute asthma exacerbation or acute respiratory tract infection during the 4 weeks prior to screening, and treatment with long-acting inhaled or systemic anticholinergics or systemic (oral or intravenous) corticosteroids within 4 weeks prior to screening.

### Study end points

All study end points were assessed as a response, defined as the difference from baseline (randomisation, Visit 2) at the end of each of the three 4-week treatment periods. The primary efficacy end point was peak FEV_1_ within 3 hours post-dosing (peak FEV_1(0–3h)_). Secondary end points included trough FEV_1_, FEV_1_ area under the curve within 3 hours post-dosing (AUC_(0–3h)_), peak forced vital capacity within 3 hours post-dosing (FVC_(0–3h)_), trough FVC, FVC AUC_(0–3h)_ and pre-dose morning and evening peak expiratory flow (PEF). Additional end points included individual FEV_1_ measurements over 3 hours post-dosing and mean forced expiratory flow 25–75% of the FVC at 4 weeks. ACQ-7 and Standardised Paediatric Asthma Quality of Life Questionnaire (PAQLQ[S]) were used to assess asthma control and quality of life, respectively.

### Assessments

Lung function assessments were performed and vital signs assessed at Visits 1–5. Lung function assessments were performed at 30 minutes, 1 hour, 2 hours and 3 hours after inhalation of study medication at screening, at the end of the 4-week run-in period and at the end of each 4-week treatment period. Patients recorded twice-daily PEF values and details of asthma symptoms, quality of life and use of rescue medication using the Asthma Monitor® AM3® device (Care Fusion, Höchberg, Germany), combining an electronic peak flow meter and electronic diary, which were reviewed by the investigator at the start of each clinic visit. Mean PEF measurements were determined in the last week of each treatment period to avoid carry-over of previous treatment effects. ACQ-7 and PAQLQ(S) data were collected during Visits 1–5 and 2–5, respectively. Adverse events (AEs) were recorded at every visit.

### Statistical analyses

Assuming a standard deviation of 280 mL for within-patient differences in peak FEV_1(0–3h)_, a sample size of 64 completer patients would be required using a full crossover design to detect a treatment difference of 100 mL for peak FEV_1(0–3h)_ based on a two-sample *t*-test with 80% power and a probability of type I error of 2.5%. Using the equation n = 3*m/2, it was calculated that 96 patients would be required for the incomplete block design used in this study. It was therefore estimated that a sample of approximately 104 patients completing the study would be sufficient for the planned statistical analyses, allowing for a drop-out rate of 8%, as observed in a similar study in adolescent patients [22].

The primary efficacy analysis was performed using the full analysis set, defined as all randomised patients who were treated with at least one dose of study medication, had baseline data and had at least one on-treatment efficacy measurement after a 4-week treatment period. Superiority of treatment with tiotropium Respimat® over placebo Respimat® was tested in a sequential hierarchical fashion at the level of α = 0.025 (one-sided) using a mixed model repeated measures analysis, with ‘treatment’ and ‘period’ as fixed effects and ‘patient’ as a random effect. The study baseline value for the end point was included in the statistical model as a covariate. Adjusted mean values, treatment contrasts, 95% confidence intervals and p values were calculated. Secondary end points were also analysed using the full analysis set and a mixed model repeated measures analysis. The treated set was used for evaluation of safety and was defined as all randomised patients who received at least one dose of study medication.

## Results

In total, 101 patients were randomised to receive study treatment. Seventy-six patients received tiotropium Respimat® 5 μg, 74 received tiotropium Respimat® 2.5 μg, 75 received tiotropium Respimat® 1.25 μg and 76 received placebo Respimat® (Additional file 1: Figure S1). One hundred patients completed all three treatment periods, with one patient discontinuing from the study prematurely (consent withdrawn for non-AE-related reasons during the first 4-week treatment period while receiving tiotropium Respimat® 5 μg); this patient was excluded from the full analysis set.

### Baseline demographics and disease characteristics

Most patients were male (68.3%), with a mean age of 8.8 years and a mean duration of asthma of 4.5 years (Table 1). Only 5.9% of patients had been exposed to household/second-hand smoking. Approximately two-thirds of patients had concomitant diseases at screening (63.4%), the most common being allergic rhinitis (53.5%).

**Table 1:** **Baseline demographics and disease characteristics (treated set)**

	**Total**
Patients, n (%)	101 (100)
Male	69 (68.3)
Female	32 (31.7)
Race, n (%)	
White	101 (100)
Age (years), mean ± SD	8.8 ± 1.7
6–8 years, n (%)	37 (36.6)
9–11 years, n (%)	64 (63.4)
Weight (kg), mean ± SD	34.2 ± 10.5
Height (cm), mean ± SD	138.9 ± 12.2
BMI (kg/m^2^), mean ± SD	17.4 ± 3.2
Smoking exposure, n (%)	
No exposure	95 (94.1)
Exposure to household/second-hand smoking	6 (5.9)
Duration of asthma (years), mean ± SD	4.5 ± 2.3
<1 year’s duration, n (%)	6 (5.9)
1–< 3 years’ duration, n (%)	18 (17.8)
≥3 years’ duration, n (%)	77 (76.2)

BMI, body mass index; SD, standard deviation.

During the 3 months prior to screening, all patients received treatment with ICS and 36.6% were also treated with a LABA, while 45.5% had taken additional leukotriene modifiers. At the time of randomisation (Visit 2), all patients were taking ICS, with 45.5% of patients also receiving leukotriene modifiers. LABAs were not permitted during the run-in or treatment periods.

Patients’ baseline asthma characteristics are summarised in Table 2. At screening, mean pre- and post-bronchodilator FEV_1_ values (± standard deviation: 1.539 ± 0.385 L, 1.909 ± 0.469 L) were 79.7% and 98.9% of predicted normal, respectively. Mean reversibility with bronchodilator use (% of pre-bronchodilator) was 370 ± 171 mL (24.6%). At baseline, mean FEV_1_ was 1.640 ± 0.386 L (85.4% of predicted normal), with 29.7% of patients having an FEV_1_ value >90% of predicted normal.

**Table 2:** **Disease characteristics measured during reversibility testing and at baseline (treated set)**

	**Reversibility testing**					
	**Pre-bronchodilator** ^**a**^		**Post-bronchodilator** ^**b**^		**Baseline pre-dose** ^**c**^	
	**Mean**	**SD**	**Mean**	**SD**	**Mean**	**SD**
FEV_1_						
Predicted normal, L	1.928	0.416				
Actual, L	1.539	0.385	1.909	0.469	1.640	0.386
Actual, % predicted normal	79.661	8.142	98.908	11.024	85.392	10.711
Reversibility, mL^d^			370	171		
Reversibility, % of pre-bronchodilator^e^			24.578	12.106		
Variation, %^f^					7.60	12.43
FVC	2.233	0.519				
Predicted normal, L	2.059	0.567	2.341	0.611		
Actual, L	92.266	13.339	105.080	15.560	2.127	0.545
Actual, % predicted normal	75.830	9.623	82.404	8.817	95.844	15.067
FEV_1_/FVC, %					78.109	9.467
PEF, L/min					220	56
ICS maintenance dose, μg (budesonide or equivalent dose)					282.2	85.8

^a^Measured 10 minutes prior to inhalation of two puffs of salbutamol (100 μg per puff) at screening (Visit 1); ^b^Measured 15–30 minutes after inhalation of two puffs of salbutamol (100 μg per puff) at screening (Visit 1); ^c^Measured 10 minutes prior to inhalation of study medication at baseline (Visit 2); ^d^Calculated as FEV_1_ post-bronchodilator – FEV_1_ pre-bronchodilator; ^e^Calculated as 100 × (FEV_1_ post-bronchodilator/FEV_1_ pre-bronchodilator) – 1; ^f^Calculated as 100 × (FEV_1_ at baseline/pre-bronchodilator FEV_1_ at screening) – 1. FEV_1_, forced expiratory volume in 1 second; FVC, forced vital capacity; ICS, inhaled corticosteroids; PEF, peak expiratory flow; SD, standard deviation.

### Efficacy

**Figure 2:**
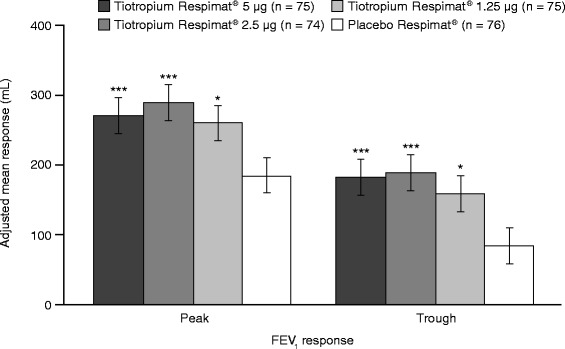
**Peak FEV**_**1(0–3h)**_ **and trough FEV**_**1**_ **responses after 4 weeks of treatment (full analysis set).** Adjusted for ‘treatment’, ‘period’, ‘patient’ and ‘baseline’. *p < 0.05; ***p < 0.001 versus placebo Respimat®. FEV_1_, forced expiratory volume in 1 second; peak FEV_1(0–3h)_, peak forced expiratory volume in 1 second within 3 hours post-dosing.

For the primary efficacy end point, statistically significant differences in peak FEV_1(0–3h)_ response after 4 weeks of treatment were observed for each tiotropium Respimat® dose group versus placebo Respimat® (Figure 2). The adjusted mean differences between tiotropium Respimat® 5 μg, 2.5 μg and 1.25 μg versus placebo Respimat® were 87 mL (p = 0.0002), 104 mL (p < 0.0001) and 75 mL (p = 0.0011), respectively. There was no dose-dependent response observed in patients treated with tiotropium Respimat®, with only minor, non-statistically significant differences between the different doses in peak FEV_1(0–3h)_ response after 4 weeks of treatment.

**Figure 3:**
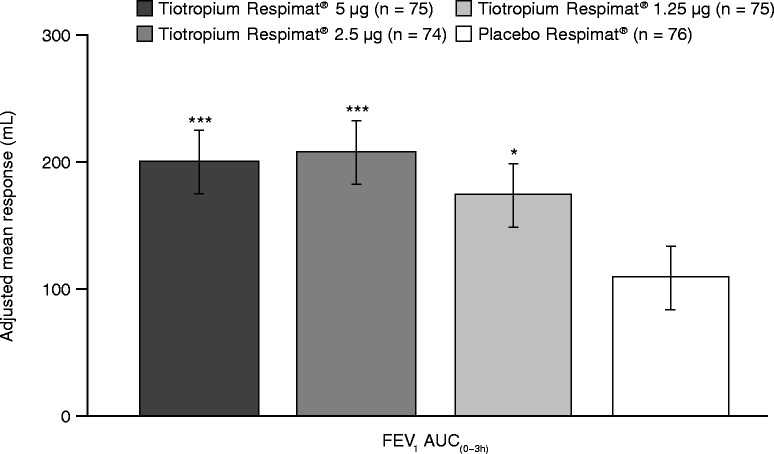
**FEV**_**1**_ **AUC**_**(0–3h)**_ **response after 4 weeks of treatment (full analysis set).** Adjusted for ‘treatment’, ‘period’, ‘patient’ and ‘baseline’. *p < 0.05; ***p < 0.001 versus placebo Respimat®. AUC_(0–3h)_, area under the curve within 3 hours post-dosing; FEV_1_, forced expiratory volume in 1 second.

**Figure 4:**
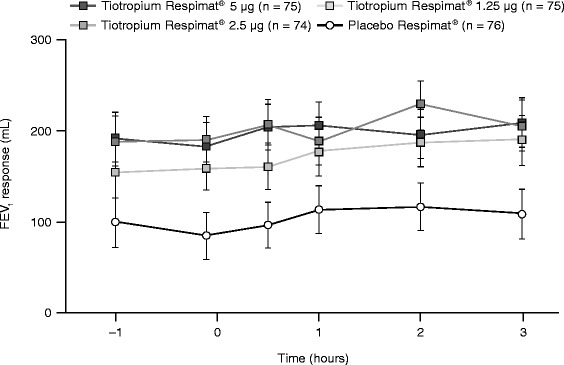
**FEV**_**1**_ **response over 3 hours post-dosing (full analysis set).** FEV_1_, forced expiratory volume in 1 second.

Tiotropium Respimat® also improved secondary and additional efficacy end points, including trough FEV_1_ response, FEV_1_ AUC_(0–3h)_ response and FEV_1_ response over 3 hours post-dosing. A statistically significant difference in adjusted mean trough FEV_1_ response was observed for each tiotropium Respimat® dose group versus placebo Respimat® (Figure 2): 5 μg = 98 mL (p < 0.0001), 2.5 μg = 105 mL (p < 0.0001) and 1.25 μg = 75 mL (p = 0.0023). A statistically significant difference in adjusted mean FEV_1_ AUC_(0–3h)_ response was also observed for each tiotropium Respimat® dose group versus placebo Respimat® (Figure 3): 5 μg = 91 mL (p < 0.0001), 2.5 μg = 99 mL (p < 0.0001) and 1.25 μg = 68 mL (p = 0.0013). The FEV_1_ responses with all doses of tiotropium Respimat® were significantly superior to those with placebo Respimat® at all time points up to 3 hours post-dosing (Figure 4).

Although peak FVC_(0–3h)_, trough FVC and FVC AUC_(0–3h)_ responses were improved on tiotropium Respimat® therapy, the improvements were statistically significant only for FVC AUC_(0–3h)_ response with the 2.5 μg dose (p = 0.0383).

**Figure 5:**
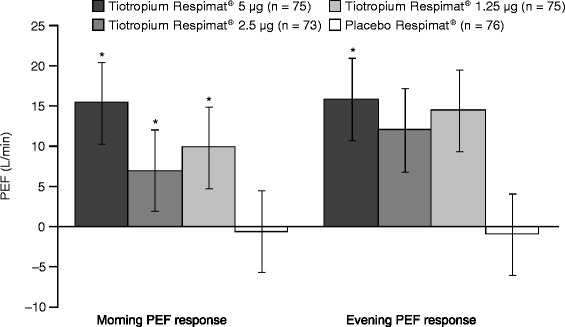
**Morning and evening PEF response after 4 weeks of treatment (full analysis set).** Adjusted for ‘treatment’, ‘period’, ‘patient’ and ‘baseline’. *p < 0.05 versus placebo Respimat®. PEF, peak expiratory flow.

Increases from baseline in morning and evening PEF responses were seen after 4 weeks for all tiotropium Respimat® dose groups. A statistically significant improvement in adjusted mean morning PEF response was observed for all three tiotropium Respimat® doses (5 μg = 16 L/min [p = 0.0036], 2.5 μg = 13 L/min [p = 0.0215] and 1.25 μg = 15 L/min [p = 0.0061]), with a statistically significant improvement in adjusted mean evening PEF response of 17 L/min (p = 0.0024) also observed for the 5 μg dose, when compared with placebo Respimat® (Figure 5).

Statistically significant improvements in adjusted mean forced expiratory flow 25–75% response were observed at all time points up to 3 hours post-dosing for all tiotropium Respimat® dose groups versus placebo Respimat®: 5 μg = 318 mL/sec, 2.5 μg = 319 mL/sec and 1.25 μg = 296 mL/sec at 3 hours post-dosing (all p *<* 0.0001). No dose-dependent response was observed, and there were no significant differences between the three tiotropium Respimat® dose groups.

Although not statistically significant, numerical improvements in asthma control and quality of life were observed following treatment with tiotropium Respimat®, compared with placebo Respimat® (ACQ-7 adjusted mean response: 5 μg = −0.088, 2.5 μg = −0.120 and 1.25 μg = −0.057; total PAQLQ[S] adjusted mean response: 5 μg = 0.091, 2.5 μg = 0.029 and 1.25 μg = 0.024).

### Safety and tolerability

The incidence of AEs experienced while receiving study treatment was comparable across the three tiotropium Respimat® dose groups and the placebo Respimat® treatment group, with events reported for approximately 10% of patients in each group (Table 3). No deaths, serious AEs, AEs leading to discontinuation of study medication, drug-related AEs or pre-specified significant AEs were reported during the study. Only one patient prematurely discontinued study medication, due to consent withdrawal for non-AE-related reasons. All other types of AE were reported in less than 3% of patients and no individual AE was reported in more than two patients in any treatment group.

**Table 3:** **Adverse events reported by more than one patient in any treatment period (treated set)**

	**Patients, n (%)**			
	**Tiotropium Respimat** **®** **5 μg**	**Tiotropium Respimat** **®** **2.5 μg**	**Tiotropium Respimat** **®** **1.25 μg**	**Placebo Respimat** **®**
	**(n = 76)**	**(n = 74)**	**(n = 75)**	**(n = 76)**
Patients with any adverse event	7 (9.2)	7 (9.5)	7 (9.3)	8 (10.5)
Asthma	2 (2.6)	2 (2.7)	1 (1.3)	1 (1.3)
Bronchitis	2 (2.6)	1 (1.4)	1 (1.3)	1 (1.3)
Headache	1 (1.3)	1 (1.4)	2 (2.7)	0
Nasopharyngitis	0	0	2 (2.7)	2 (2.6)
Pharyngitis	1 (1.3)	1 (1.4)	0	1 (1.3)
Rhinitis	0	2 (2.7)	0	1 (1.3)
Cough	0	0	0	2 (2.6)
Influenza	1 (1.3)	1 (1.4)	0	0
Purulence	0	0	1 (1.3)	0
Respiratory tract infection	0	0	0	1 (1.3)
Urinary tract infection	0	0	0	1 (1.3)
Viral upper respiratory tract infection	1 (1.3)	0	0	0
Stomatitis	0	0	0	1 (1.3)
Chest pain	1 (1.3)	0	0	0
Contusion^a^	0	0	0	1 (1.3)
Skeletal injury	0	0	0	1 (1.3)

^a^Contusion of the lower jaw. Treatment + 30 days.

## Discussion

In the present study, once-daily tiotropium Respimat® add-on to medium-dose ICS, with or without a leukotriene modifier, improved lung function in children with symptomatic asthma. For the primary end point, statistically significant improvements in peak FEV_1(0–3h)_ response after 4 weeks of treatment were observed for all tiotropium Respimat® dose groups versus placebo Respimat®.

Analyses of secondary and additional efficacy end points also generally demonstrated the superiority of all tiotropium Respimat® doses tested, with statistically significant improvements in trough FEV_1_, FEV_1_ AUC_(0–3h)_ and peak FEV_1_ at all time points up to 3 hours post-dosing observed with all doses of tiotropium Respimat®, compared with placebo Respimat®. The observed improvements in FVC were generally not significantly different from those observed with placebo Respimat®, which is an expected observation given the age of this patient population.

PEF monitoring is an important tool for measuring airway changes, particularly in patients who may not accurately perceive their worsening symptoms [25]. PEF results, which represent a weekly average of daily values, may provide more reliable data compared with FEV_1_ measurements, which represent a single value taken on 1 day in a clinic outside of a patient’s real-life setting. Data from the study presented here demonstrate that all doses of tiotropium Respimat® were superior to placebo Respimat® for morning PEF. For evening PEF, which represents a true 24-hour value, the tiotropium Respimat® 5 μg dose also showed superiority when compared with placebo Respimat®, and demonstrated higher values than the 2.5 μg and 1.25 μg doses.

With regard to patient-reported outcomes, a positive trend for improvements in ACQ-7 and PAQLQ(S) scores was observed in this study following treatment with all three doses of tiotropium Respimat®. Additional analyses from parallel-group trials of longer duration and with larger patient numbers are required to further investigate the effect of tiotropium Respimat® on asthma control and quality of life in children with symptomatic asthma.

The study presented here demonstrates that once-daily tiotropium Respimat® add-on to medium-dose ICS, with or without a leukotriene modifier, has safety and tolerability that are comparable with those of placebo Respimat® in children aged 6–11 years with symptomatic asthma. This parallels and further reinforces the data in adult patients, where once-daily tiotropium Respimat® was shown to have similar safety and tolerability when compared with placebo Respimat® in patients with symptomatic asthma on ICS with or without a LABA [20,26,27].

Although the comparison between the tiotropium Respimat® doses was descriptive only, we note that there was no clear dose-dependent response seen for either the primary or any of the secondary or additional efficacy end points. The results of Phase II studies in adult and adolescent patients with asthma have clearly demonstrated a greater response with the 5 μg dose. In these Phase II studies, the once-daily doses of 10 μg, 5 μg, 2.5 μg or 1.25 μg all improved lung function and were well tolerated, with the 5 μg dose achieving the greatest bronchodilation [17,18,22,28]. The long-term clinical efficacy and safety of tiotropium Respimat® 5 μg have been demonstrated in two large Phase III studies in adult patients with symptomatic asthma receiving ICS plus LABA [20].

The Respimat® SoftMist™ inhaler may provide advantages over pressurised metered-dose inhalers and dry-powder inhalers, particularly in the treatment of children with asthma. The increased aerosol production time with the SoftMist™ inhaler may benefit young patients with low inspiratory capacity or poor timing of inhalation to actuation, although correct technique remains important [29]. A single device with once-daily dosing may also improve patient adherence, which is notably poor in children and adolescents [11].

It should be noted that this study has some methodological limitations. The incomplete-crossover design means that all patients did not receive all study treatments; however, this study design reduces inter-patient variability and the number of patients required to reach statistical power, with the lack of washout between treatments promoting patient compliance. The short study duration meant that the focus was on assessment of lung function, and did not allow for a full assessment of asthma symptom control or exacerbation rate.

The data presented in this manuscript encourage and warrant future, large Phase III trials in paediatric patients to confirm these results and to examine the impact of tiotropium Respimat® add-on therapy on long-term efficacy, safety and tolerability. Additional studies will help to determine where tiotropium Respimat® will fit in future treatment guidelines, particularly in relation to high-dose ICS maintenance therapy with or without a LABA.

## Conclusion

This first study of tiotropium Respimat® in children with symptomatic asthma has shown that tiotropium Respimat® add-on to medium-dose ICS, with or without a leukotriene modifier, is efficacious and has comparable safety and tolerability with placebo Respimat®. A larger Phase III study is warranted to confirm these promising initial findings.

## Additional file

Additional file 1: Figure S1
**Enrolment, randomisation and study completion. AE, adverse event.**

## Abbreviations

ACQ-7: Seven-question Asthma Control Questionnaire
AE: Adverse event
AUC_(0–3h)_: Area under the curve within 3 hours post-dosing
BMI: Body mass index
FEV_1_: Forced expiratory volume in 1 second
FVC: Forced vital capacity
ICS: Inhaled corticosteroids
LABA: Long-acting β_2_-agonist
PAQLQ(S): Standardised Paediatric Asthma Quality of Life Questionnaire
Peak FEV_1(0–3h)_: Peak forced expiratory volume in 1 second within 3 hours post-dosing
PEF: Peak expiratory flow
QD: Once-daily
SD: Standard deviation
