# Erratum to: A randomised dose-ranging study of tiotropium Respimat® in children with symptomatic asthma despite inhaled corticosteroids

**DOI:** 10.1186/s12931-015-0290-7

**Published:** 2015-10-20

**Authors:** Christian Vogelberg, Petra Moroni-Zentgraf, Migle Leonaviciute-Klimantaviciene, Ralf Sigmund, Eckard Hamelmann, Michael Engel, Stanley Szefler

**Affiliations:** University Hospital Carl Gustav Carus, Technical University of Dresden, Fetscherstraße 74, 01307 Dresden, Germany; Boehringer Ingelheim Pharma GmbH & Co. KG, Ingelheim am Rhein, Germany; Vilnius University Hospital, Vilnius, Lithuania; Boehringer Ingelheim Pharma GmbH & Co. KG, Biberach an der Riss, Germany; Evangelisches Krankenhaus Bielefeld, Bielefeld, Germany; Department of Pediatrics, Children’s Hospital of Colorado and the University of Colorado Denver School of Medicine, Aurora, Colorado USA

## Erratum

Following publication of our article [1] we identified an error in Fig. 5, where results were inadvertently switched between morning and evening PEF responses. The corrected Fig. 5 is provided in this erratum. The PEF results provided in our original article text remain correct.

**Fig. 5:**
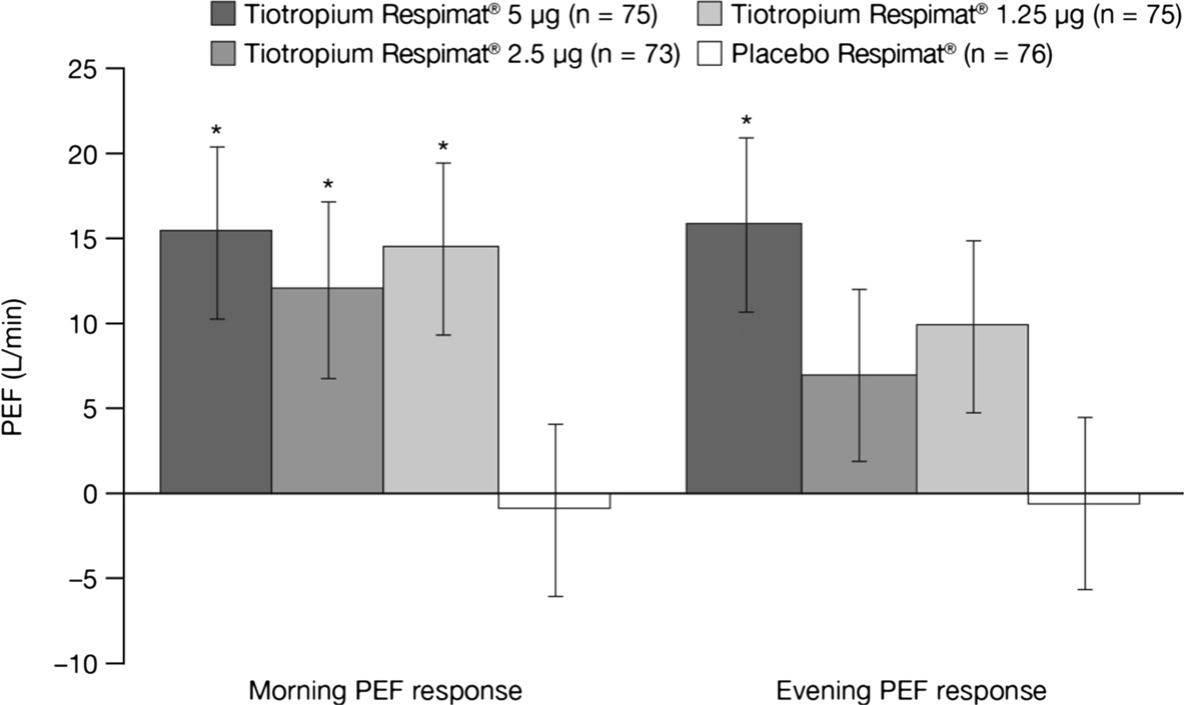
Morning and evening PEF response after 4 weeks of treatment (full analysis set). Adjusted for ‘treatment’, ‘period’, ‘patient’ and ‘baseline’. *p < 0.05 versus placebo Respimat®. PEF, peak expiratory flow
