# Supplementary figures and images for: A randomised dose-ranging study of tiotropium Respimat® in children with symptomatic asthma despite inhaled corticosteroids
Source: Respir Res. 2015 Feb 7;16(1):20. doi: 10.1186/s12931-015-0175-9 (PMC4331449; doi:10.1186/s12931-015-0175-9)

Figure S1

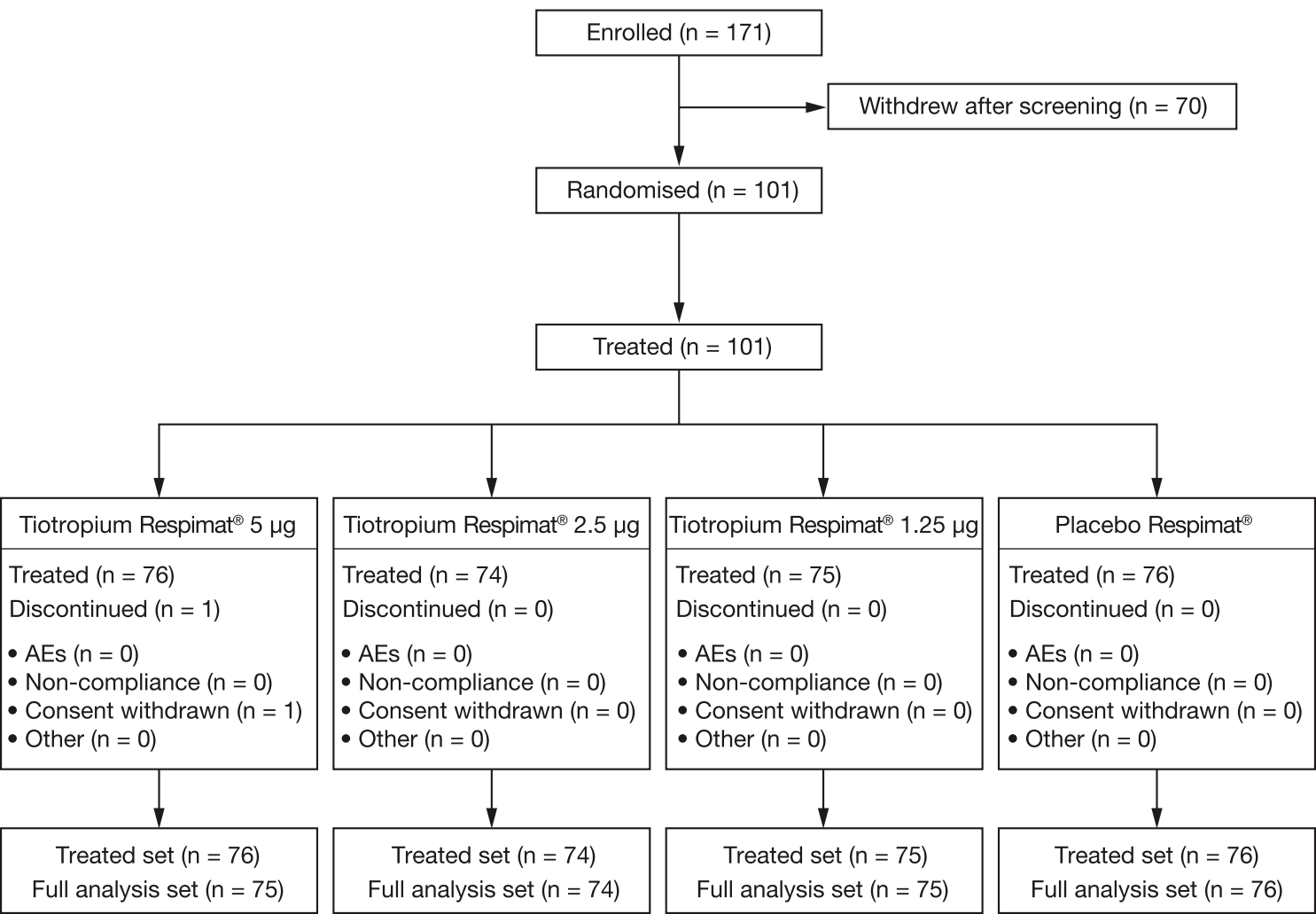

Supplement: Additional file 1: Figure S1 — Enrolment, randomisation and study completion. AE, adverse event. [file 12931_2015_175_MOESM1_ESM.pdf]
